# Supercritical CO_2_ Plant Extracts Show Antifungal Activities against Crop-Borne Fungi

**DOI:** 10.3390/molecules27031132

**Published:** 2022-02-08

**Authors:** Katja Schoss, Nina Kočevar Glavač, Jasna Dolenc Koce, Sabina Anžlovar

**Affiliations:** 1 Department of Pharmaceutical Biology, Faculty of Pharmacy, University of Ljubljana, Aškerčeva 7, SI-1000 Ljubljana, Slovenia; katja.schoss@ffa.uni-lj.si (K.S.); nina.kocevar.glavac@ffa.uni-lj.si (N.K.G.); 2 Department of Biology, Biotechnical Faculty, University of Ljubljana, Jamnikarjeva 101, SI-1000 Ljubljana, Slovenia; sabina.anzlovar@bf.uni-lj.si

**Keywords:** antifungal activity, growth inhibition, plant extract, supercritical CO_2_ extraction, *Botrytis cinerea*, *Chamomilla recutita*

## Abstract

Fungal infections of cultivated food crops result in extensive losses of crops at the global level, while resistance to antifungal agents continues to grow. Supercritical fluid extraction using CO_2_ (SFE-CO_2_) has gained attention as an environmentally well-accepted extraction method, as CO_2_ is a non-toxic, inert and available solvent, and the extracts obtained are, chemically, of greater or different complexities compared to those of conventional extracts. The SFE-CO_2_ extracts of *Achillea millefolium*, *Calendula officinalis*, *Chamomilla recutita*, *Helichrysum arenarium*, *Humulus lupulus*, *Taraxacum officinale*, *Juniperus communis*, *Hypericum perforatum*, *Nepeta cataria*, *Crataegus* sp. and *Sambucus nigra* were studied in terms of their compositions and antifungal activities against the wheat- and buckwheat-borne fungi *Alternaria alternata*, *Epicoccum nigrum*, *Botrytis cinerea*, *Fusarium oxysporum* and *Fusarium poae.* The *C. recutita* and *H. arenarium* extracts were the most efficacious, and these inhibited the growth of most of the fungi by 80% to 100%. Among the fungal species, *B. cinerea* was the most susceptible to the treatments with the SFE-CO_2_ extracts, while *Fusarium* spp. were the least. This study shows that some of these SFE-CO_2_ extracts have promising potential for use as antifungal agents for selected crop-borne fungi.

## 1. Introduction

Fungi are an integral part of the natural environment, and although they have many positive roles, such as for industrial exploitation in the pharmaceutical, cosmetic, agricultural and food industries [1,2,3,4,5], they are also major contributors to food spoilage. Pathogenic fungi are responsible for up to a 20% loss of global crop yields yearly, which includes the loss of 125 million tons of the five most cultivated food crops, while 10% of crops are destroyed during post-harvest [6]. Fungicides and fungistatics are therefore widely used chemicals that are typically of synthetic origin, and these kill parasitic fungi or their spores, or inhibit their growth, respectively. Resistance to many of the effective fungicides and fungistatics has gradually spread in pathogen populations, which has motivated the search for new alternatives [7]. Furthermore, tightening the regulations on chemical use and the modern perspective on harmful residues has led to renewed focus on natural products, such as plant extracts and essential oils, and their use as antifungal agents [8].

The antimicrobial activities of plant extracts are attributed to the presence of secondary metabolites, such as alkaloids, phenols, flavonoids and terpenoids, and several studies have shown inhibitory activities of extracts against different post-harvest fungi [9]. The composition of plant extracts is, however, dependent on various factors, such as the plant species, the part of the plant used and the growing conditions, which include soil, climate and time of harvest. Furthermore, the choice of an extraction method is extremely important to provide comparable final products that maintain the primary composition of the plant source [10].

New technological approaches to extract these substances are gaining increased attention across a variety of research fields, such as the use of supercritical fluid extraction (SFE). This interest is due to the clean and environmentally-friendly approaches available, with no associated waste treatments of toxic solvents necessary, and the moderate extraction times needed. Moreover, SFE allows for the precise control over the process parameters, which generally results in high selectivity and extraction yields. The SFE of essential oils is of particular interest, as this avoids the more traditional hydrodistillation that can cause chemical alterations to the products, mainly due to temperature- and oxidation-dependent reactions [11,12].

In practice, SFE is mainly performed using carbon dioxide (CO_2_), due to its low critical pressure (74 bar) and temperature (32 °C), and its non-toxicity, non-flammability, inertness and low cost. Additionally, CO_2_ is easily removed from the extracts, and the products obtained with SFE-CO_2_ have a ‘generally recognized as safe’ (i.e., GRAS) status according to the U.S. Food and Drug Administration [11,13].

The objective of the present study is to determine the antifungal activities of SFE-CO_2_ extracts of 11 plant species: yarrow (*Achillea millefolium*), marigold (*Calendula officinalis*), chamomile (*Chamomilla recutita*), sandy everlasting (*Helichrysum arenarium*), hops (*Humulus lupulus*), dandelion (*Taraxacum officinale*), juniper (*Juniperus communis*), St. John’s wort (*Hypericum perforatum*), catnip (*Nepeta cataria*), hawthorn (*Crataegus* sp.) and elderberry (*Sambucus nigra*). For these plant species, previous reports on the antifungal activities of their extracts are available, although different extraction methods and fungal species were used. Therefore, we selected five crop-borne fungi that were previously isolated from wheat and buckwheat grain [14,15]: *Alternaria alternata* and *Epicoccum nigrum* as saprophytes, and *Botrytis cinerea*, *Fusarium poae* and *Fusarium oxysporum* as pathogenic fungi. The inhibition of fungal growth was used to evaluate the antifungal activities of the selected SFE-CO_2_ extracts, for which the chemical compositions of volatile compounds were determined by gas chromatography–mass spectrometry (GC–MS). The aim is to determine the most efficacious of these plant extracts, and to determine whether all of these fungi are equally susceptible to these SFE-CO_2_ extracts.

## 2. Results and Discussion

### 2.1. Compositions of the SFE-CO_2_ Extracts

The compositions of the volatile compounds identified in the SFE-CO_2_ extracts are presented in Table 1 for those with retention times <70 min and peak heights >60,000 units. The compounds with relative peak areas >5% were recognized as the main components, and are discussed below in relation to previously published studies. On this basis, the SFE-CO_2_ extract of common juniper had the highest number of detected volatile compounds (77), followed by elderberry (55), chamomile (46), marigold (45), yarrow (31), catnip (29), St. John’s wort (26), sandy everlasting (21), hops (21), dandelion (18) and hawthorn (2).

The SFE-CO_2_ extracts of the juniper fruit studied by Barjaktarović et al. [16] also showed a great diversity of compounds, which depended on the pressures and extraction times used. Their extract with the most similar results (separation conditions 80 bar, 40 °C) to the present study contained germacrene D (16.07%) and germacrene B (13.11%) among its main components. This correlates with the present study, where germacrene D (18.46%) and germacrene B (6.63%) were also present, together with sandaracopimarinal (7.90%) and 2 unknown compounds with relative peak areas of 7.61% and 5.30%.

To the best of our knowledge, the present study is the first to focus on the composition of a SFE-CO_2_ extract of elderberry flowers. The 5 main compounds detected here were ethyl palmitate (12.46%), n-pentacosane (11.09%), n-tricosane (10.76%), n-heneicosane (10.09%) and an unidentified peak (18.75%). Similarly, heneicosane (18.8%), tricosane (17.3%) and pentacosane (10.3%) were confirmed as the main compounds in an elderberry flower essential oil obtained by hydrodistillation [17].

The main components of the chamomile flower extract were α-bisabolol oxide A (24.40%), tonghaosu (21.36%), pentacosane (14.08%), bisabolol oxide B (8.32%) and β-farnesene (6.56%). The same compounds were described previously in chamomile essential oils obtained by hydrodistillation [18], while bisabolol oxide A (50.42%), bisabolol oxide B (16.88%) and β-farnesene (1.53%) were also identified in an SFE-CO_2_ flower extract by Reverchon et al. (separation conditions 90 bar, 40 °C) [19].

In the marigold flower extracts obtained by Petrović et al. [20], α-cadinol (22.41%), γ-cadinene (7.24%) and δ-cadinene (19.87%) were found among the main constituents (separation conditions 200 bar, 40 °C). The present study identified pentacosane (14.49%), δ-cadinene (12.45%), nonacosane (7.46%), γ-cadinene (7.42%), α-cadinol (7.39%) and heneicosane (5.32%).

The genus *Achillea* is a group of difficult-to-distinguish species and subspecies. Thus, the compositions of its essential oils have often been very variable, although with some characteristic components, such as chamazulene, camphor, sabinene, α- and β-pinene, eucalyptol and caryophyllene [21]. The last 3 of these compounds were also detected in the present study, as caryophyllene (12.17%), eucalyptol (6.36%) and β-pinene (5.48%), in addition to pentacosane (6.75%) and an unknown compound (10.60%).

The 4 main compounds in the catnip extract were eucalyptol (30.28%), caryophyllene (9.89%), germacrene D (33.01%) and caryophyllene oxide (5.19%). Caryophyllene (2.1%) and caryophyllene oxide (0.1%) were also identified in a study of a hydrodistilled essential oil of flowering catnip [22], although the major compounds were reported to be β-nepetalactone (55–59%) and α-nepetalactone (30–31%).

The main constituents of the St. John’s wort SFE-CO_2_ extract were caryophyllene oxide (12.67%), heneicosane (7.44%), phytol (7.39%), an unknown component (7.02%) and caryophyllene (5.70%), while Smelcerovic et al. [23] also reported caryophyllene oxide (1.40%) and phytol (7.13%) in an SFE-CO_2_ extract (separation conditions 100 bar, 40 °C), and caryophyllene oxide (0.94%), heneicosane (14.41%) and phytol (2.44%) in a subcritical CO_2_ extract.

Poor chemical characterization was achieved for the sandy everlasting extract. Only 37.92% of the components were identified, with γ-curcumene at 12.74%. However, the composition determined here was not comparable with that reported for an essential oil, which was composed of α-cedrene, α-selinene, cyclosativene, α-ylangene and limonene [24].

For the SFE-CO_2_ extracts of hops, Nagybákay et al. [25] reported α-humulene and β-humulene (7.96% and 6.31%, respectively), β-pinene (7.02), β-mircene (6.23%) and α-selinene (5.49%) as the major constituents (separation conditions 370 bar, 43 °C), which does not compare with the results of the present study. Here, the volatile compounds in the extract as analyzed by GC–MS remained largely undefined, as we were only able to identify 3.3% of the compounds detected.

Dandelion is usually used to make extracts from the roots or leaves, while flower extracts are not common, although they were studied here. The previously determined content of volatile compounds in dandelion flowers was low (0.08% essential oil) and included nonadecane and hexadecane [26], which were also detected in the present study (1.63% and 28.16%, respectively). However, the composition of the dandelion extract remained largely undefined, as 60.71% of the compounds detected were not identified.

In the hawthorn extract, only 2 volatile components were detected, tricosane (46.23%) and pentacosane (53.77%), which is partially comparable to a study on *Crataegus monogyna* flower essential oil, which contained 12% to 17% tricosane [27].

The analysis and comparisons of the bioactive compounds obtained in extractions of plant materials is generally very complex. Extracts of the same plant species obtained by the same method of extraction are difficult to compare, as their compositions depend on various factors, including climatic conditions, geographic location, harvest time and plant part. It is even more difficult to compare extracts prepared by different extraction methods, such as water or steam distillation versus supercritical extraction.

The pressure, temperature and extraction time were shown to be the main extraction parameters defining the composition of SFE-CO_2_ extracts, as well as the extraction yield [16,20,23,25]. Higher pressures typically lead to higher yields; however, in terms of volatile compounds, higher pressures typically lead to a significant co-extraction of nonvolatile compounds [16,25,28]. Nagybákay et al. [25] showed that in GC–MS analyses the relative peak intensities of total monoterpenes increased with an increase in pressure, while total sesquiterpenes decreased. At the level of an individual plant, it is therefore reasonable to perform detailed extraction optimization, to find a suitable compromise between extraction yield and the content of target bioactive compounds.

Based on the results presented here, SFE-CO_2_ extraction can be seen to be a promising extraction method for the production of new plant extracts with potentially new or enhanced biological activities. Key known constituents typically present in extracts obtained by conventional hydrodistillation are generally retained, while additional chemical complexity is achieved due to the specific properties of CO_2_ as a solvent. Along with the advantages of SFE-CO_2_ as an environmentally-acceptable extraction method that can provide good selectivity and yields, which is highly desirable at an industrial as well as a laboratory scale, SFE-CO_2_ extracts have great potential for exploring new activities and mechanisms of action, and, finally, they also have diverse applications.

### 2.2. Antifungal Activities of the SFE-CO_2_ Extracts

The antifungal activities of the SFE-CO_2_ extracts obtained were assessed by comparing the growth of fungal colonies on media without and with the extracts added (i.e., versus control samples with solvent only). In a preliminary experiment, the fungi were grown on media with 20%, 10% and 2% concentrations of the extracts, and the 10% concentration was selected as the most suitable for further testing. These 10% extracts significantly inhibited the growth of most of the fungal species tested (Table 2).

The chamomile extract has the highest antifungal activity, as it inhibits the growth of most of these fungi by 80% to 100%, followed by the extracts of sandy everlasting, hops, juniper, yarrow, marigold, elderberry, catnip, St. John’s wort, dandelion and hawthorn, in decreasing order (Table 2). Among all of the fungi, on average the inhibition of the growth of *B. cinerea* is the greatest (45–100%), followed by *E. nigrum*, *A. alternata*, *F. poae* and *F. oxysporum*, in decreasing order (Table 2).

The results of the present study show that the SFE-CO_2_ extracts of these plant species have antifungal activities, which also confirms their previously reported effects where different fungal species, plant materials and extraction methods were used. For example, Glišić et al. [29] evaluated the antifungal activity of a juniper fruit SFE-CO_2_ extract, and strong fungicidal activity was observed against *Candida* sp. and dermatophytes, although the extract composition was different, as it was reported to contain α-pinene, sabinene and myrcene as the main compounds. For chamomile, essential oils have been shown to have antibacterial activities against *Listeria monocytogenes*, *Escherichia coli*, *Salmonella enterica* and *Staphylococcus aureus* [30], and antifungal activities against *Microsporum canis*, *M. gypseum*, *Trichophyton tonsurans*, *T. mentagrophytes* and *T. rubrum* [31]. Methanol and ethanol extracts from marigold flowers were shown to have antibacterial (*Bacillus subtilis*, *B. cereus*, *B. pumilis*, *Pseudomonas aeruginosa*, *E. coli*, *S. aureus*, *Klebsiella aerogenes*, *K. pneumoniae* and *Enterococcus faecalis*) and antifungal (*Candida albicans*, *C. krusei*, *C. glabrata*, *C. parapsilosis*, *Aspergillus flavus*, *A. fumigatus*, *A. niger* and *Exophiala dermatitidis*) activities [32]. In a study by Aydin and Sevindik [33], an essential oil of a yarrow subspecies *A. millefolium* subsp. *millefolium* was shown to be efficacious against *C. albicans*, *C. tropicalis*, *C. parapsilosis* and *Saccharomyces cerevisiae*. St. John’s wort extracts were also shown to have antibacterial (*P. aeruginosa*, *S. aureus*, *S. oxford*, *S. mutans*, *S. sanguis*, *E. coli*, *P. vulgaris*, *S. pyogenes* and *H. pylori*) and antifungal (*M. gypseum*, *T. rubrum*, *A. flavus*, *C. lunata*, *F. vasiinfectum*, *A. niger*, *F. oxysporum*, *P. canescens*, *H. sativum* and *F. graminearum*) activities [34]. Antimicrobial activities of a dandelion extract were shown against *Streptococcus mutans*, *S. pyogenes*, *S. pneumonia*, *S. aureus* and *P. aeruginosa* in a study by Mir et al. [35].

As the greatest inhibition of fungal growth was achieved with the chamomile and sandy everlasting extracts here (Supplemental Figures S1 and S2), their antifungal activities were further evaluated by concentration-dependence testing. In general, higher extract concentrations resulted in greater fungal growth inhibition, although the fungi showed different susceptibilities to extract treatments (Table 3 and Table 4). The least sensitive fungus (i.e., *F. oxysporum*) was inhibited by the high extract concentrations, while the growth of the more sensitive fungus (i.e., *B. cinerea*) was even inhibited by the two lowest concentrations. These dose-related data demonstrate that the inhibition of fungal growth is indeed caused by the extracts.

As noted before, the chamomile SFE-CO_2_ extract had the greatest inhibitory effects on these crop-borne fungi. Many studies have previously confirmed antimicrobial activities for chamomile essential oils. For example, a study of the in vitro antifungal activities of different essential oils against pathogenic seed-borne fungi revealed that chamomile essential oil was the most active [36]. The main mechanism of this antifungal activity of chamomile essential oil has been indicated as being due to formation of a superoxide anion and peroxide, and the associated oxidative stress [37]. In combination with antimicrobial agents fluconazole and nystatin, chamomile essential oil showed synergistic and additive inhibitory effects against a clinical *Candida* strain [37]. Further, a chamomile essential oil was compared with α-bisabolol oxide A, β-farnesene, chamazulene and α-bisabolol (the four main compounds in the SFE-CO_2_ chamomile extract here) for in vitro antioxidant activities using the DPPH free radical scavenging microdilution assay. The highest inhibitory activity was observed for chamazulene, followed by α-bisabolol oxide A, the essential oil and β-farnesene [38]. In a study by Forrer et al. [39], α-bisabolol showed antimicrobial activity against *Solobacterium moorei*, a Gram-positive bacterium associated with halitosis (i.e., bad breath). De Lucca et al. [40] investigated the fungicidal properties of synthetic α-bisabolol, which resulted in nearly a 98% loss of viability for the germinating conidia of *A. flavus*, *A. fumigatus*, *A. niger*, *A. terreus*, *F. oxysporum*, *F. solani* and *F. verticillioides*. Therefore, the high antifungal activity of the chamomile SFE-CO_2_ extract appears to be related to its high content of α-bisabolol (Table 1).

This SFE-CO_2_ extract of chamomile flowers completely inhibited the growth of the pathogenic *B. cinerea* as well as the saprophytic *E. nigrum* (Table 2 and Table 3). Due to this broad and potent antifungal activity, the chamomile flower SFE-CO_2_ extract would be a suitable candidate for potential application as an environmentally-friendly agent for seed treatment. Other plant extracts here also showed relatively high antifungal activities, especially sandy everlasting and hops, although we considered their range of inhibition (approx. 50–80%) to be too low for potential applications.

As noted earlier, *B. cinerea* was the most susceptible fungal species to the actions of these SFE-CO_2_ extracts. The chamomile and yarrow extracts were the most potent, as they completely inhibited the growth of *B. cinerea*, while the majority of the other extracts (except for St. John’s wort extract) also showed their highest antifungal activities against *B. cinerea*.

*Botrytis* spp. is a group of fungi with a broad host range and geographic distribution. These have a significant economic impact on horticulture, as they can cause diseases, such as grey mold, leaf blight, blossom blight and stem rot [41]. *B. cinerea* infects more than 200 plant species and causes grey mold that is visible on the surface as fluffy grey mycelia [42]. Unfortunately, there are no adequate and safe treatments to control this fungal disease, as it can counteract a wide range of plant defense chemicals [42]. To some extent, it can be limited in the field by a combination of fungicides. However, the misuse of fungicides can lead to fungicide resistance and environmental pollution [43]. Biological control of the fungus is therefore a better choice.

High antifungal activity against *B. cinerea* has been reported previously for SFE-CO_2_ root extracts of *Echinacea angustifolia* [44], which also belongs to the Asteraceae family, as are the chamomile and yarrow in the present study. Growth inhibition of *B. cinerea* by the chamomile extract was concentration dependent, and with the 6.25% extract the inhibition was still close to 80% (Table 3). In contrast, an essential oil of chamomile at a concentration of 250 ppm showed no inhibition of mycelial growth of *B. cinerea* [45]. Behshti et al. [46] reported that chamomile essential oil only weakly inhibited the growth of *B. cinerea*, compared to complete inhibition by their anise oil at concentrations of 800 µL/L. In a study by Šernaite et al. [47], SFE-CO_2_ extracts of cinnamon and pimento showed high in vitro antifungal activities against two different isolates of *B. cinerea*, from strawberry and apple fruits. Despite the high in vitro antifungal activity, the extracts they studied were not equally effective when applied to the apples. Complete inhibition by the SFE-CO_2_ chamomile and yarrow extracts in the present study shows that these two extracts have potential for use as antifungal agents against *B. cinerea*.

The other pathogenic fungi tested in the present study were from the genus *Fusarium*. Interestingly, for the majority of the SFE-CO_2_ extracts, the *Fusarium* spp. were the least susceptible. The chamomile extract showed the lowest inhibition against *F. oxysporum*, while the sandy everlasting extract showed the lowest inhibition against *F. poae* (Table 2). The elderberry extract showed a distinctly different species-specific inhibition of *Fusarium* growth, whereby *F. poae* was inhibited by nearly 80%, while *F. oxysporum* was inhibited by less than 20% (Table 2). The hops extract showed a similar but opposite species-specific growth inhibition of *Fusarium* spp., with 70% inhibition of *F. oxysporum* and 20% inhibition of *F. poae*. Differential sensitivities of *Fusarium* spp., and even isolates of the same species, were reported by Krzysko-Łupicka et al. [48]. They investigated the efficacy of some commercially available essential oils on isolates of common central European parasitic fungal species of *Fusarium* obtained from infected wheat grains. *Fusarium* isolates from the German sample were generally more sensitive than those from the Polish sample. The susceptibility of individual Fusarium species varied, and regardless of the origin of the isolates, their susceptibilities were as follows (from most to least sensitive): *F. culmorum*, *F. graminearum*, *F. poae*, *F. avenaceum* and *F. oxysporum* [48].

## 3. Materials and Methods

### 3.1. Plant Material and Extraction Procedure

Dried herbs of commercially available wild-grown plants from the region of Bihač, Bosnia and Herzegovina, were obtained from IME Insol (Slovenia) (Table 5). The samples were ground and extracted with a BBES 2.0 extraction system (Waters, Milford, MA, USA) equipped with a 10 L extraction vessel and three consecutive 2 L collection vessels (CV). Extractions were performed in the extraction vessel under 200–300 bar and 40–50 °C, depending on the plant material (Table 5). Supercritical CO_2_ and the extract mixture were separated in three collection vessels, CV1, CV2 and CV3, under the following separation conditions: 130 bar, 45 °C (CV1), 72 bar, 35 °C (CV2) and 50 bar, 30 °C (CV3) (Table 5). A detailed description of the BBES, as well as a schematic flow diagram of the BBES, is available in [49]. The plant extracts from CV2 and CV3 were combined, mixed and stored at 4 °C for further analysis. The extracts from CV1, which contained waxes and highly non-polar compounds, were discarded, and were therefore not analyzed.

### 3.2. GC–MS Analysis

Samples of the SFE-CO_2_ extracts were diluted in n-hexane (Suprasolv, Merck, Germany) (1 mg/mL), transferred to GC-vials, and analyzed using a GC–MS system (GCMS-QP2010 Ultra; Shimadzu Corporation, Kyoto, Japan) equipped with an MS column (Rxi-5Sil; 30 m × 0.25 mm i.d.; film thickness, 0.25 μm; Restek Corporation, Bellefonte, PA, USA). The samples were injected (1 μL) using an autosampler at a 1:100 split ratio. The GC conditions were: injector temperature, 250 °C; helium (99.99%) as the carrier gas; and flow rate, 1 mL/min. The initial temperature was 50 °C, which was raised to 250 °C over 5 min, with a total analysis time of 91.67 min. MS conditions were: electrospray ionization mode; ionization voltage, 70 eV; ion source temperature, 200 °C; m/z range, 40.0–400.0; and scanning frequency, 5 Hz.

The identification of the compounds was based on a comparison of their mass spectra and retention indices with those of the synthetic compounds spectral library of the National Institute of Standards and Technology (NIST11) [50], and the Flavors and Fragrances of Natural and Synthetic Compounds spectral library (FFNSC2) [51].

### 3.3. Antifungal Activity

The antifungal activities of the SFE-CO_2_ extracts were tested against five molds: *A. alternata*, *E. nigrum*, *B. cinerea*, *F. oxysporum* and *F. poae*. These fungi were previously isolated from wheat and buckwheat grain and identified by molecular methods [14,15]. The fungal endophytes *A. alternata* and *E. nigrum* are saprophytic, whereas *B. cinerea*, *F. oxysporum* and *F. poae* are considered to be plant pathogens.

The inhibitory effects of the SFE-CO_2_ extracts on radial growth of the fungal mycelia were tested according to the method described in our previous study [52]. First, 10% extracts were prepared by mixing 0.1 g of each SFE-CO_2_ extract and 0.9 mL 70% ethanol (Merck, Germany), with each extract stirred on a vibrating mixer until dissolved. The elderberry and dandelion extracts were dissolved in acetone (Merck, Germany).

A volume of 50 µL of 10% SFE-CO_2_ extracts was spread over 2% (*w/v*) potato dextrose agar (Biolife, Italy) in a Petri dish (2r = 90 mm) using a Drigalski spatula. Disks of fungal mycelia (2r = 5 mm) were cut from the margins of 7-day-old fungal cultures and aseptically inoculated by placing them in the center of a fresh plate with an extract. Control samples were prepared at the same time, with 70% ethanol or acetone and without the extracts. The fungal colonies were incubated at room temperature (23 ± 2 °C) in the dark for 7 days. Mycelial growth was assessed on day 7 after inoculation. The plates were photographed with a digital camera (EOS 1000D; Canon, Tokyo, Japan) and the areas (cm^2^) of the fungal colonies were calculated using image processing software (ImageJ). The inhibition of the fungal growth was expressed as the proportion (%) of growth reduction, as calculated according to Equation (1), by Anžlovar et al. [8]:

Inhibition (%) = (AC − AT)/AC × 100(1)

where AC is the area of mycelial growth of the control colonies, and AT is the area of mycelial growth of the treated colonies. Three replicates (N = 3) were carried out for the controls and for each treatment.

In addition, the 2 most active extracts (chamomile and sandy everlasting) were further tested at 50%, 25%, 12.5%, 6.25% and 3.125% concentrations.

### 3.4. Statistical Analysis

For the antifungal activities, three fungal colonies were measured per treatment. Growth inhibition data were analyzed statistically to calculate the mean values and standard errors, and the treatments were compared with *t*-tests (MS Excel). The level of statistical significance was set at *p* < 0.05.

The GC–MS identification of compounds in SFE-CO_2_ extracts was performed for a single sample per extract.

## 4. Conclusions

Research of new antifungal agents of a natural origin has become increasingly focused in environmentally-friendlier extraction methods, such as the extractions of plant materials using supercritical CO_2_. SFE-CO_2_ extracts generally have promising potential for use as antifungal agents for selected crop-borne fungi. The present study shows the most significant growth inhibition of pathogenic *B. cinerea* by chamomile and yarrow SFE-CO_2_ extracts. Other combinations of extracts and fungi were less promising for antifungal treatments. To assess the potential of these extracts as antifungal preparations, a broader range of harmful fungi should be tested in further studies.

Based on the results presented, we conclude that SFE-CO_2_ extraction is a promising method for the production of plant extracts with antifungal activities. Constituents typically present in hydrodistilled essential oils are generally retained, while a new chemical complexity is achieved on account of CO_2_ as a solvent. For the extracts with the most significant antifungal activity, detailed phytochemical characterization should be performed in further studies, using HPLC and/or LC-MS methods.

## Figures and Tables

**Table 1:** Gas chromatography–mass spectrometry compositions of volatile compounds in the SFE-CO_2_ extracts according to their relative peak intensity (RPI), as determined in this study. The compounds were identified based on their mass spectra and retention indices (RI; Db, database RI; Ms, measured RI). Unidentified compounds are presented as numbers according to their four most intensive mass ion peaks.

		RI		RPI
Species	Compound	Db	Ms	(%)
*Juniperus communis*	α-pinene	933	932	2.71
	sabinene	972	972	1.80
	myrcene	991	990	1.64
	terpinen-4-ol	1184	1180	2.41
	β-elemene	1390	1389	1.64
	caryophyllene, (E)	1424	1419	3.54
	α-humulene	1454	1455	3.03
	germacrene D	1480	1481	18.46
	bicyclogermacrene	1497	1495	1.81
	γ-cadinene	1512	1513	1.12
	δ-cadinene	1518	1518	1.21
	germacrene B	1557	1559	6.63
	81 (100), 43 (43), 41 (22), 123 (21)			7.61
	oplopanone	1738	1732	1.57
	abietatriene	2052	2058	1.69
	93 (100), 81 (94), 79 (84), 41 (81)			2.28
	sandaracopimarinal	2187	2183	7.90
	larixol	2263	2257	1.47
	81 (100), 109 (72), 107 (71), 55 (68)			5.3
	dehydro-abietol	2371	2359	1.16
	81 (100), 41 (73), 93 (72), 107 (72)			1.38
*Sambucus nigra*	neophytadiene	1836	1838	1.44
	n-nonadecane	1900	1902	4.78
	ethyl-palmitate	1993	1994	12.46
	n-heneicosane	2100	2102	10.09
	ethyl-linoleate	2164	2159	10.06
	79 (100), 67 (63), 95 (60), 93 (55)			18.75
	ethyl-oleate	2173	2173	4.04
	ethyl-stearate	2198	2194	1.68
	n-docosane	2200	2202	2.26
	n-tricosane	2300	2303	10.76
	ethyl-eicosanoate	2394	2395	1.07
	n-tetracosane	2400	2403	1.35
	n-pentacosane	2500	2503	11.09
*Chamomilla recutita*	β-farnesene, (E)	1452	1451	6.56
	α-bisabolol oxide B	1655	1652	8.32
	α-bisabolone oxide A	1682	1678	2.69
	epi-alpha-bisabolol	1679	1683	1.97
	hernianin	1720	1715	2.07
	chamazulene	1728	1726	2.80
	α-bisabolol oxide A	1748	1746	21.71
	tonghaosu, (Z)	1883	1874	18.39
	tonghaosu, (E)	1895	1887	2.97
	228 (100), 199 (100), 171 (81), 43 (81)			1.01
	228 (100), 185 (90), 43 (85), 213 (83)			1.05
	n-tricosane	2300	2300	2.41
	244 (100), 43 (99), 159 (65), 91 (58)			2.25
	n-pentacosane	2500	2500	14.08
*Calendula officinalis*	α-humulene	1454	1456	1.02
	γ-muurolene	1478	1476	1.13
	germacrene D	1480	1482	1.04
	207 (100), 43 (97), 161 (81), 93 (67)			1.28
	α-muurolene	1497	1499	2.3
	γ-cadinene	1512	1514	7.42
	δ-cadinene	1518	1519	12.45
	α-cadinene	1538	1538	1.5
	epi-alpha-cadinol	1640	1643	3.64
	t-muurolol	1645	1645	2.26
	cadin-4-en-10-ol	1659	1656	7.39
	oplopanone	1738	1733	1.23
	n-nonadecane	1900	1903	3.33
	n-heneicosane	2100	2103	5.32
	43 (100), 58 (91), 55 (67), 57 (57)			1.53
	55 (100), 79 (66), 91 (45), 41 (42)			1.88
	n-nonacosane	2305	2303	7.46
	79 (100), 43 (74), 55 (71), 80 (67)			1.5
	79 (100), 43 (71), 55 (65), 41 (60)			6.78
	n-tetracosane	2400	2403	1.06
	n-pentacosane	2500	2503	14.49
*Achillea millefolium*	sabinene	972	972	4.26
	β-pinene	978	977	5.48
	eucalyptol	1032	1032	6.36
	camphor	1149	1147	1.53
	borneol	1173	1172	1.91
	terpinen-4-ol	1184	1181	2.54
	α-terpineol	1195	1195	1.79
	caryophyllene, (E)	1424	1420	12.17
	α-humulene	1456	1454	1.22
	germacrene D	1480	1482	5.42
	α-zingiberene	1496	1496	1.54
	caryophyllene oxide	1587	1583	5.39
	43 (100), 108 (64), 93 (56), 67 (30)		1682	2.35
	137 (100), 84 (74), 119 (73), 41 (62)		1688	1.39
	neophytadiene	1836	1839	1.13
	109 (100), 110 (71), 69 (50), 43 (44)			4.42
	phytol	2106	2111	1.59
	69 (100), 81 (64), 41 (51), 93 (32)			2.24
	43 (100), 55 (70), 41 (56), 81 (53)			3.45
	95 (100), 81 (81), 55 (61), 73 (57)			1.22
	n-nonacosane	2305	2303	2.21
	43 (100), 213 (34), 228 (33), 185 (23)			2.82
	231 (100), 232 (17), 246 (11), 121 (10)			10.6
	57 (100), 43 (91), 82 (88), 96 (71)			2.59
	67 (100), 81 (84), 55 (81), 95 (60)			1.55
	73 (100), 355 (59), 281 (46), 221 (42)			2.05
	n-pentacosane	2500	2504	6.75
	2-ethylhexylbisphthalic acid	2531	2529	1.09
	55 (100), 228 (84), 213 (54), 172 (41)			1.07
*Nepeta cataria*	eucalyptol	1032	1031	30.28
	α-terpineol	1195	1194	2.34
	β-bourbonene	1382	1383	1.67
	caryophyllene, (E)	1424	1419	9.89
	α-humulene	1454	1455	1.88
	germacrene D	1480	1481	33.01
	caryophyllene oxide	1587	1581	5.19
	phytol	2106	2108	1.42
	n-pentacosane	2500	2501	2.67
*Hypericum perforatum*	43 (100), 57 (69), 71 (47), 41 (36)			1.94
	α-pinene	933	933	1.71
	43 (100), 57 (33), 41 (31), 85 (27)			1.28
	43 (100), 45 (51), 41 (30), 85 (29)			1.29
	57 (100), 71 (67), 43 (47), 41 (37)			1.62
	57 (100), 43 (98), 71 (69), 41 (39)			1.76
	57 (100), 43 (89), 71 (57), 85 (51)			3.31
	caryophyllene, (E)	1424	1420	5.7
	β-farnesene, (E)	1452	1453	2.37
	germacrene D	1480	1475	2.38
	caryophyllene oxide	1587	1582	12.67
	tetradec-2-enal, (trans)	1673	1678	3.4
	n-nonadecane	1900	1902	3.17
	n-heneicosane	2100	2102	7.44
	phytol	2106	2109	7.39
	69 (100), 43 (79), 41 (76), 109 (48)			3.24
	n-nonacosane	2305	2302	4.25
	69 (100), 41 (57), 43 (56), 398 (38)			3.66
	69 (100), 43 (98), 123 (83), 41 (68)			4.87
	43 (100), 69 (91), 41 (71), 71 (42)			7.02
	73 (100), 355 (62), 147 (45), 221 (43)			2.68
	69 (100), 41 (69), 43 (51), 193 (50)			4.51
	n-pentacosane	2500	2502	3.13
	69 (100), 43 (75), 41 (65), 113 (50)			4.57
	57 (100), 85 (48), 69 (44), 41 (43)			2.37
	41 (100), 69 (99), 57 (62), 43 (58)			2.27
*Helichrysum arenarium*	α-pinene	933	933	4.19
	geranyl acetate, (cis)	1361	1359	3.1
	italicene	1410	1406	1.25
	caryophyllene, (E)	1424	1420	3.23
	55 (100), 133 (99), 43 (98), 41 (84)			1.2
	γ-curcumene	1482	1478	12.74
	α-curcumene	1480	1481	2.62
	β-selinene	1492	1489	3.73
	α-selinene	1501	1496	2.17
	205 (100), 83 (45), 55 (24), 79 (16)			1.27
	43 (100), 145 (78), 218 (36), 157 (31)			7.36
	43 (100), 145 (81), 200 (67), 160 (30)		1359	6.48
	219 (100), 234 (95), 43 (62), 201 (57)			3.91
	181 (100), 43 (68), 236 (58), 165 (42)			9.94
	145 (100), 200 (90), 43 (88), 160 (49)			7
	219 (100), 248 (50), 177 (32), 233 (30)			2.16
	165 (100), 221 (36), 264 (19), 69 (15)			6.84
	83 (100), 82 (78), 55 (48), 57 (35)			3.21
	83 (100), 82 (54), 55 (42), 57 (22)			10.64
	73 (100), 355 (61), 281 (47), 221 (47)			2.07
	n-pentacosane	2500	2503	4.89
*Humulus lupulus*	69 (100), 41 (61), 70 (15), 82 (15)			1.49
	α-humulene	1454	1456	1.38
	248 (100), 136 (85), 233 (67), 41 (51)			1.26
	69 (100), 197 (87), 41 (58), 266 (21)			2.88
	69 (100), 41 (68), 57 (49), 248 (41)			2.44
	135 (100), 69 (98), 181 (97), 105 (71)			1.29
	238 (100), 239 (63), 223 (46), 182 (46)			2.07
	182 (100), 238 (61), 277 (59), 119 (55)			1.16
	69 (100), 41 (53), 197 (36), 57 (29)			1.45
	69 (100), 275 (87), 41 (84), 263 (48)			39.79
	289 (100), 69 (92), 41 (82), 233 (49)			8.51
	289 (100), 69 (82), 41 (72), 277 (50)			29.98
*Taraxacum officinale*	caryophyllene, (E)	1424	1419	1.45
	neophytadiene	1836	1836	3.05
	phytone	1841	1840	1.54
	n-nonadecane	1900	1900	1.63
	methyl-hexadecanoate	1925	1924	2.01
	methyl-linoleate	2093	2089	2.06
	n-heneicosane	2100	2100	28.16
	phytol	2106	2108	8.96
	84 (100), 43 (43), 41 (41), 57 (35)			1.76
	tributyl-citrate acetate	2243	2246	1.31
	n-nonacosane	2305	2300	6.62
	69 (100), 81 (57), 41 (40), 93 (39)			1.84
	231 (100), 232 (17), 246 (12), 121 (10)			19.3
	69 (100), 81 (82), 93 (49), 41 (48)			1.49
	57 (100), 82 (93), 43 (82), 96 (68)			4.18
	73 (100), 355 (66), 221 (52), 281 (48)			2.03
	n-pentacosane	2500	2500	3.92
	59 (100), 58 (88), 43 (76), 71 (46)			8.69
*Cratageus* sp.	n-tricosane	2300	2305	46.23
	n-pentacosane	2500	2498	53.77

**Table 2:** Antifungal activities of the 10% SFE-CO_2_ extracts. Data are the means ± standard error (N = 3).

SFE-CO_2_ Extract	Inhibition of Growth of Fungal Mycelia (%)				
	*A. alternata*	*E. nigrum*	*F. poae*	*F. oxysporum*	*B. cinerea*
German chamomile	86.75 ± 1.67 *	100.00 ± 0.00 *	88.15 ± 7.17 *	57.61 ± 18.19 *	100.00 ± 0.00 *
Sandy everlasting	79.13 ± 2.84 *	79.70 ± 2.67 *	44.82 ± 11.06 *	72.52 ± 3.37 *	49.81 ± 8.36
Common hops	72.32 ± 7.49 *	81.18 ± 7.60 *	21.46 ± 4.38	67.10 ± 2.72 *	76.87 ± 14.47 *
Common juniper	38.89 ± 5.89 *	54.79 ± 5.80 *	46.04 ± 13.47 *	9.30 ± 4.21	67.01 ± 7.85
Yarrow	21.46 ± 4.67 *	49.54 ± 13.44 *	21.95 ± 5.05 *	6.41 ± 3.23	100.00 ± 0.00 *
Common marigold	42.02 ± 3.67 *	58.06 ± 12.28	11.19 ± 8.01	15.59 ± 4.18 *	69.57 ± 6.59 *
Black elderberry	−2.13 ± 2.09	11.36 ± 8.53	75.21 ± 2.70	18.66 ± 1.82 *	81.13 ± 3.68 *
Catnip	36.01 ± 1.54 *	41.23 ± 5.74	33.82 ± 2.84 *	21.32 ± 10.25	87.57 ± 2.19 *
St. John’s wort	14.78 ± 6.28 *	65.96 ± 15.67 *	17.05 ± 8.14 *	24.24 ± 7.72 *	−11.86 ± 3.47 *
Dandelion	−4.55 ± 0.68	7.67 ± 2.95	−1.39 ± 2.20	16.58 ± 5.65	44.76 ± 12.83
Hawthorn	21.40 ± 8.43	7.06 ±6.53	−9.65 ± 3.88	−7.37 ± 5.31	79.43 ± 5.14 *

*, *p* <0.05, significant difference vs. relevant control (*t*-tests).

**Table 3:** Concentration-dependent antifungal activities of the chamomile SFE-CO_2_ extract. Data are the means ± standard error (N = 3).

Extract	Inhibition of Growth of Fungal Mycelia (%)				
Concentration (%)	*A. alternata*	*E. nigrum*	*F. poae*	*F. oxysisporum*	*B. cinerea*
50	97.07 ± 1.10	100.00 ± 0.00	93.72 ± 5.68	77.79 ± 15.18	100.00 ± 0.00
25	94.34 ± 2.50	98.30 ± 2.94	92.35 ± 6.95	65.32 ± 5.46	100.00 ± 0.00
12.5	90.50 ± 5.81	96.03 ± 3.88	90.13 ± 7.50	68.52 ± 18.99	100.00 ± 0.00
6.25	86.36 ± 6.50	82.58 ± 10.91	80.34 ± 9.88	59.64 ± 13.64	75.62 ± 10.87
3.125	63.66 ± 5.44	61.84 ± 11.28	25.75 ± 12.38	14.76 ± 9.72	39.74 ± 18.89

**Table 4:** Concentration-dependent antifungal activities of the sandy everlasting SFE-CO_2_ extract. Data are the means ± standard error (N = 3).

Extract	Inhibition of Growth of Fungal Mycelia (%)				
Concentration (%)	*A. alternata*	*E. nigrum*	*F. poae*	*F. oxysisporum*	*B. cinerea*
50	97.95 ± 3.55	85.41 ± 7.49	77.44 ± 13.26	89.79 ± 10.11	94.66 ± 5.06
25	87.33 ± 7.10	85.25 ± 2.84	64.73 ± 9.85	77.05 ± 4.47	85.78 ± 4.29
12.5	85.11 ± 7.87	83.10 ± 4.60	56.50 ± 11.19	63.37 ± 7.60	82.16 ± 9.08
6.25	82.48 ± 11.95	83.05 ± 2.96	51.96 ± 8.76	69.85 ± 6.91	81.38 ± 4.90
3.125	82.64 ± 10.68	86.23 ± 3.93	39.27 ± 11.73	54.13 ± 0.28	80.90 ± 7.56

**Table 5:** The plants and plant parts used for the SFE-CO_2_ extraction, and the extraction parameters used.

Plant	Species	Family	Plant Part	Extraction Parameter	
				Pressure (bar)	Temperature (°C)
Yarrow	*Achillea millefolium*	*Asteraceae*	Flowering herb	250	45
Common marigold	*Calendula officinalis*	*Asteraceae*	Flower	200	45
German chamomile	*Chamomilla recutita*	*Asteraceae*	Flower	200	45
Sandy everlasting	*Helichrysum arenarium*	*Asteraceae*	Flower	230	40
Common hops	*Humulus lupulus*	*Cannabaceae*	Flower	300	40
Dandelion	*Taraxacum officinale*	*Cichoriaceae*	Flower	250	50
Common juniper	*Juniperus communis*	*Cupressaceae*	Fruit	250	45
St. John’s wort	*Hypericum perforatum*	*Hypericaceae*	Flowering herb	230	40
Catnip	*Nepeta cataria*	*Lamiaceae*	Herb	300	45
Hawthorn	*Crataegus* sp.	*Rosaceae*	Flower	300	40
Black elderberry	*Sambucus nigra*	*Sambucaceae*	Flower	300	45

Separation conditions (pressure, temperature): collection vessel 1 (130 bar, 45 °C); collection vessel 2 (72 bar, 35 °C); collection vessel 3 (50 bar, 30 °C).

## Data Availability

The data presented in this study are available in supplementary material.

## Supplementary Materials

The following are available online, Figure S1: Concentration-dependent fungal growth after treatments with chamomile SFE-CO_2_ extracts and control. Figure S2: Concentration-dependent fungal growth after treatments with sandy everlasting SFE-CO_2_ extracts and control.

## Sample Availability

Samples of the compounds are not available from the authors.
