# Supplementary material for: Supercritical CO2 Plant Extracts Show Antifungal Activities against Crop-Borne Fungi
Source: Molecules. 2022 Feb 8;27(3):1132. doi: 10.3390/molecules27031132 (PMC8838041; doi:10.3390/molecules27031132)
Supplement: Supplementary file 1 [file molecules-27-01132-s001.zip › molecules-1553448-supplementary.pdf]

molecules

MDPI

Supplement for the Article

Supercritical CO_2 Plant Extracts Show Antifungal Activities against Crop-Borne Fungi

Katja Schoss[1], Nina Kočevar Glavač[1], Jasna Dolenc Koce[2]*, Sabina Anžlovar[2]

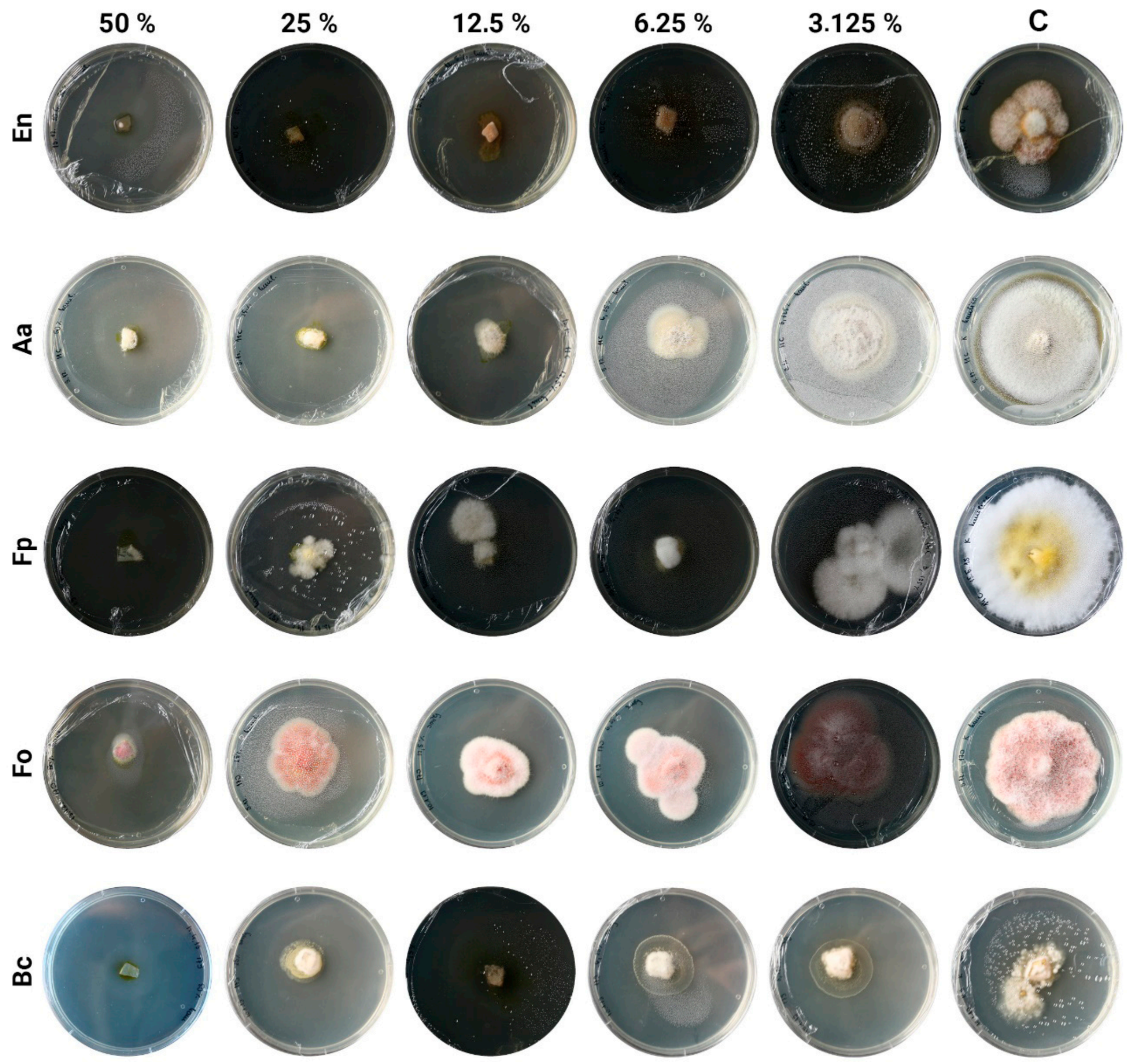

Figure S1. Concentration-dependent fungal growth after treatments with chamomile SFE-CO_2 extracts and control (C). Fungal strains are indicated with letters: En – *E. nigrum*, Aa – *A. alternata*, Fp – *F. poae*, Fo – *F. oxysporum*, Bc – *B. cinerea.*

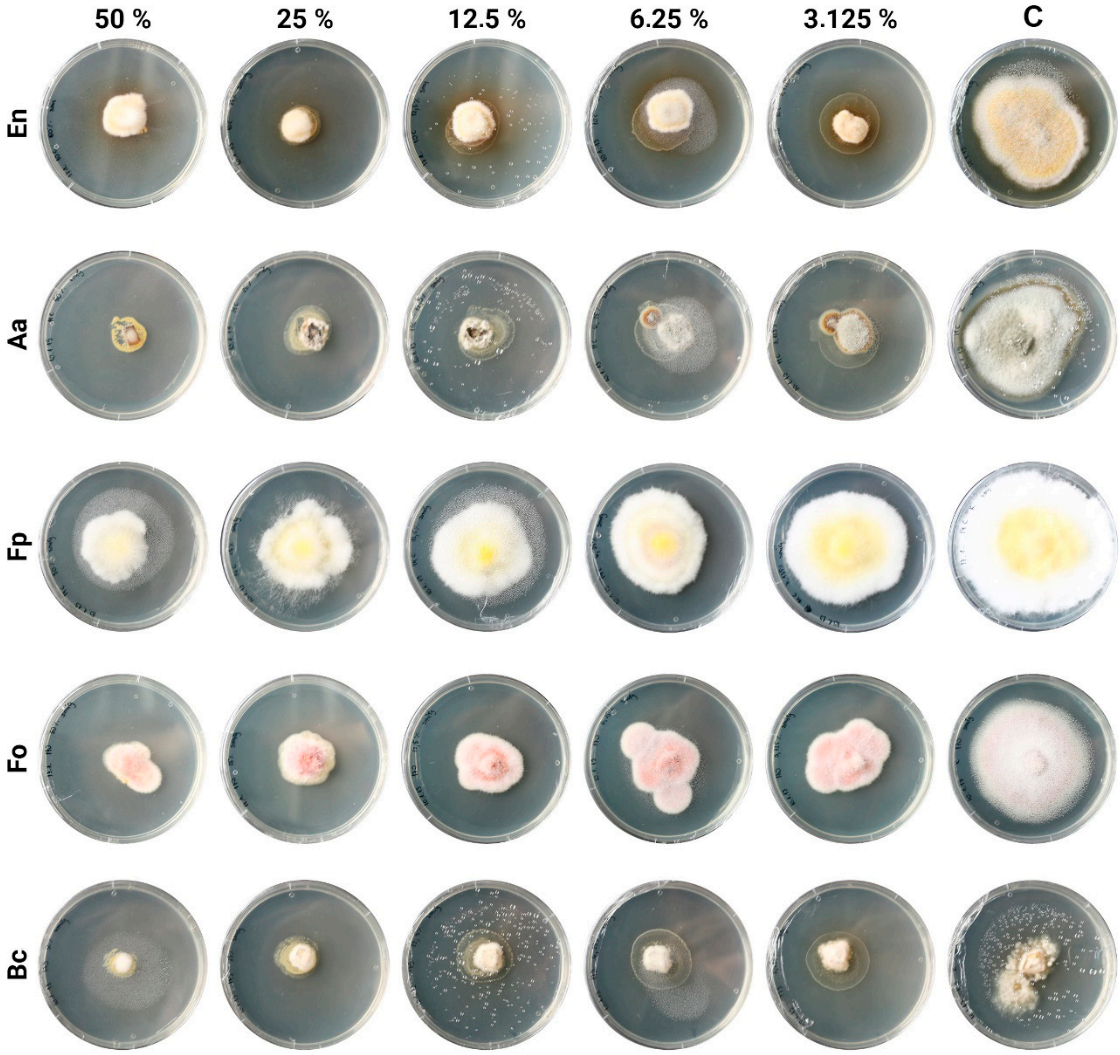

Figure S2. Concentration-dependent fungal growth after treatments with sandy everlasting SFE-CO_2 extracts and control (C). Fungal strains are indicated with letters: En – *E. nigrum*, Aa – *A. alternata*, Fp – *F. poae*, Fo – *F. oxysporum*, Bc – *B. cinerea.*
